# Transient Ischemic Attack Due to Delamination of a Steerable Guiding Sheath: A Potential Complication During Atrial Fibrillation Ablation

**DOI:** 10.19102/icrm.2026.17093

**Published:** 2026-09-15

**Authors:** Hafez Golzarian, Khalil Kanjwal, Fayaz Hakim

**Affiliations:** 1 Department of Cardiovascular Disease, HCA Houston Healthcare/University of Houston College of Medicine, Houston, TX, USA; 2 Department of Cardiac Electrophysiology, Ascension Genesys Henry Ford Health System, Grand Blanc, MI, USA

**Keywords:** Catheter ablation, delamination, Vizigo™ sheath

## Abstract

We report a case of transient ischemic attack due to a thromboembolic event secondary to delamination of the steerable guiding sheath, CARTO VIZIGO™ (J&J MedTech, New Brunswick, NJ, USA), indicating a device deficiency in structural integrity. This case illustrates an unforeseen device-related complication, emphasizing the importance of heightened vigilance for this potential complication during catheter ablations incorporating this sheath.

## Case presentation

A 65-year-old woman with symptomatic persistent atrial fibrillation failed rhythm control with amiodarone and decided to undergo catheter ablation. Her medical history was significant for hypertension and obstructive sleep apnea, with compliance with continued positive airway pressure therapy. Echocardiography showed normal left ventricular systolic function (ejection fraction, 50%–55%), mild concentric left ventricular hypertrophy (wall thickness, 1.2 cm), and a mildly dilated left atrium (left atrial volume index, 32 mL/m^2^). Stress myocardial imaging performed a year prior to the procedure showed no perfusion defects. The patient was electively brought to the electrophysiology laboratory and underwent right and left pulmonary vein isolation in pairs using radiofrequency wide antral circumferential ablation. The procedure was performed under general anesthesia, on uninterrupted anticoagulation with apixaban and with zero fluoroscopy using the CARTO™ 3 (J&J MedTech, New Brunswick, NJ, USA) electroanatomic mapping system. A single transseptal puncture was performed under intracardiac echocardiography (ICE) guidance using a SOUNDSTAR™ catheter (J&J MedTech) and the VersaCross™ radiofrequency transseptal system (Baylis Medical Technologies, Inc., Mississauga, Canada). Heparin boluses were given before transseptal puncture and throughout the procedure to maintain the activated clotting time between 300 and 350 s. Pre- and post-ablation voltage maps of the left atrium were created using a multispline, multipolar irrigated mapping catheter, Octaray™ (J&J MedTech), which has an 8-Fr outer diameter. Ablation was performed in a point-by-point contiguous fashion using a ThermoCool SmartTouch™ SF (STSF) catheter (J&J MedTech) at a power of 50 W to a target ablation index of 400–450 for the posterior wall and 500–550 for the anterior wall. A steerable, medium-curve, bidirectional guiding sheath, CARTO VIZIGO™ (J&J MedTech), was used during mapping and ablation to improve catheter stability and maneuverability (sheath inner diameter, 8.5 Fr). The catheters and sheath were connected to heparinized/saline flush lines as per the manufacturer’s recommendations. An uneventful acute pulmonary vein isolation was achieved during the first pass, and, as such, the catheter exchanges were minimal. No apparent defects were noted during catheter preparation or sheath assembly prior to their use. We also did not encounter more-than-usual resistance or difficulties during catheter exchanges. At the end of the procedure, the catheters and sheaths were removed, and hemostasis was achieved by deploying VASCADE^®^ vascular closure devices (Haemonetics, Boston, MA, USA) in the femoral venotomy sites.

The assisting technician then noted a dark mass protruding through the tip of the Vizigo™ sheath, which was initially thought to be a clot. The sheath was flushed with saline, a lump of fine filamentous plastic/fabric was retrieved, and excess blood was washed away with saline **(Figure 1)**. The end of the filament was attached to the tip of the Vizigo™ sheath and could be reclaimed like a yarn from a sweater **(Figure 2)**.

**Figure 1:**
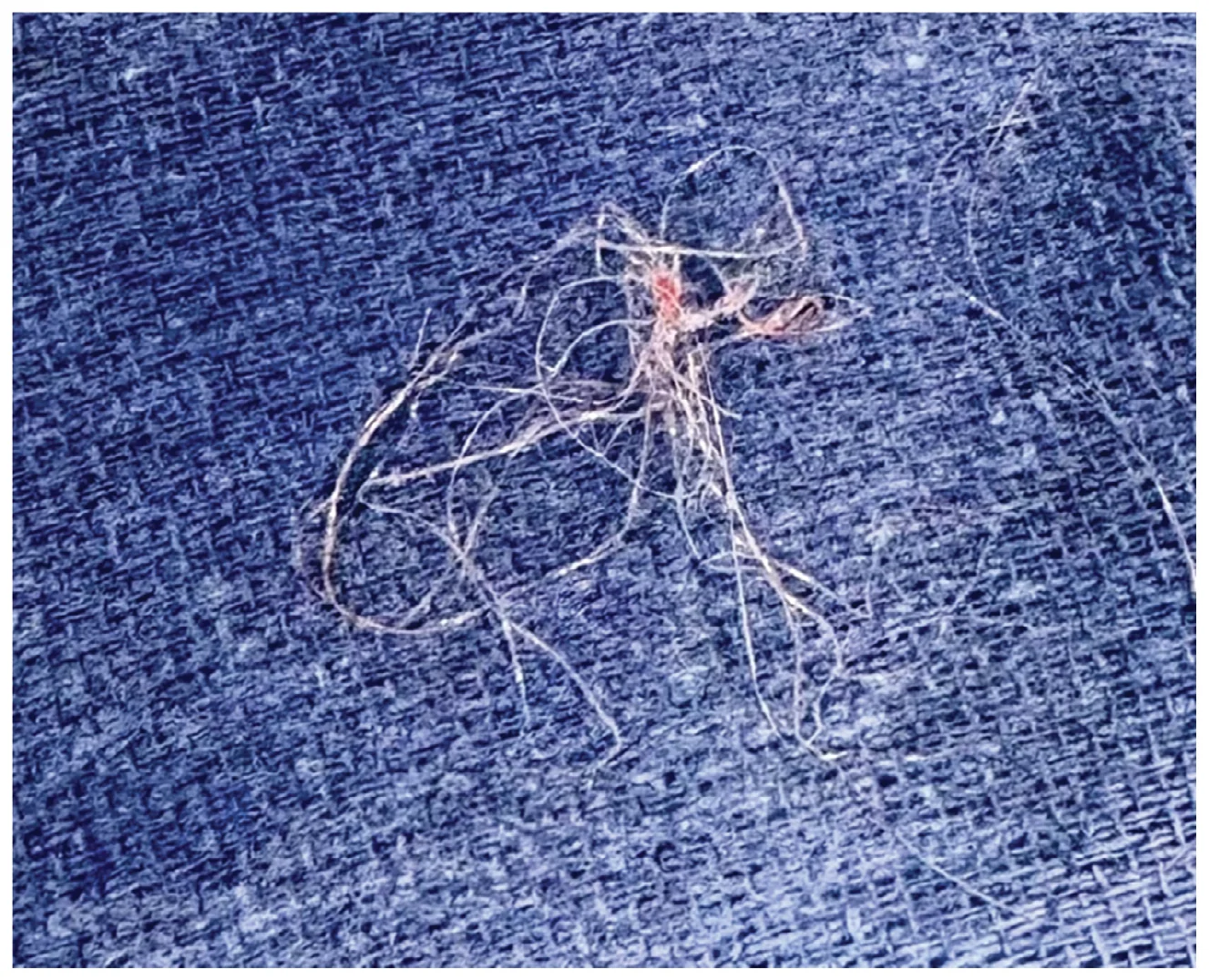
Filamentous material extracted at the conclusion of the case.

**Figure 2:**
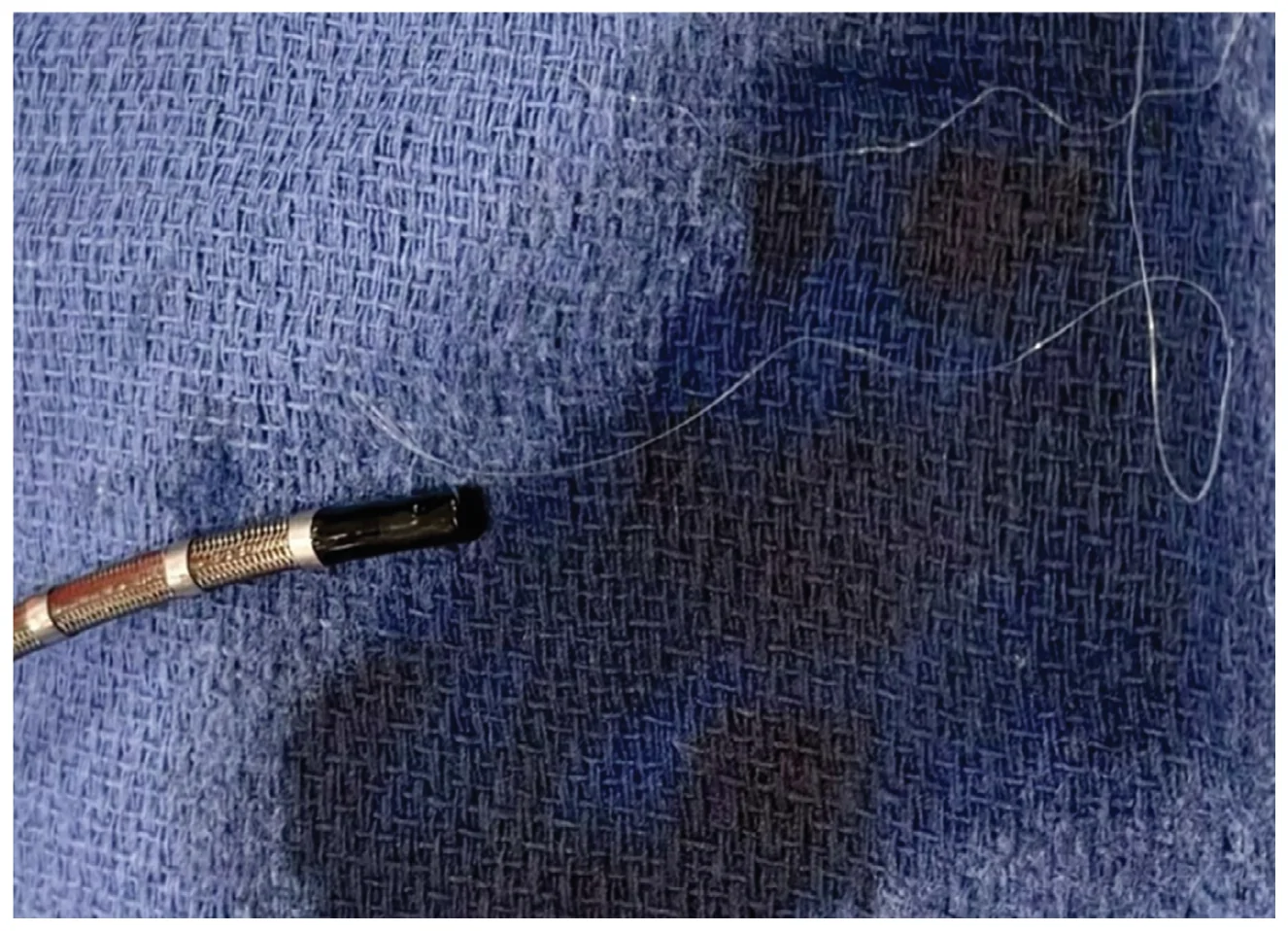
Filamentous delamination from the Vizigo™ bidirectional guiding sheath.

The STSF ablation and Octaray™ mapping catheters and the tip and hemostatic valve of the Vizigo™ sheath were examined, and no defects or breaches in integrity were noted. The Vizigo™ sheath was sent for analysis, yielding observations consistent with delamination. The patient complained of significant blurring of vision and had difficulty identifying faces with her right eye while she was being monitored in the recovery unit. With concerns of ophthalmic stroke, the stroke team was promptly alerted, and a neurological consultation was sought. Symptoms were fully resolved within 4 h. Magnetic resonance imaging of the brain revealed old subcortical lacunar infarcts but no acute pathology. Oral anticoagulation was resumed. She was admitted for overnight observation and discharged home the following morning in a stable condition. Outpatient follow-up encounters at 3, 6, and 12 months were all unremarkable.

Direct consent was obtained from the patient for the publication of this manuscript.

## Discussion

Delamination of hemodynamic and mapping catheters is rare and has been reported anecdotally in the literature.^1,2^ Although detachment of the hemostatic valve and distal electrode ring has been reported with the use of the Vizigo™ sheath during atrial fibrillation ablation,^3^ we report the first case of structural delamination of this sheath that resulted in a thromboembolic event. A breach in structural integrity with the release of fabric or plastic material has the potential to embolize and predispose to thromboembolism, as seen in our patient.

Damage to structural integrity and subsequent delamination and release of fabric from the guiding sheaths during catheter ablation can occur due to manufacturing defects, operator technique, or the size and type of equipment passed through the sheath. The CARTO Vizigo™ sheath has a braided shaft with silicone coating and polyurethane polymer to enable good torque and friction response. However, internal silicone or polytetrafluoroethylene (PTFE) liners can be damaged and peel off during catheter exchange. Although the risk of delamination is theoretically greater with reprocessed sheaths, it may even occur with brand new sheaths well within the shelf life, as seen in our case. To the best of our knowledge, this is the first reported case of Vizigo™ sheath delamination. Gatto et al. described delamination of a CARTO™ 3 multielectrode mapping catheter, PentaRay™ (J&J MedTech), with extraction of a long translucent filamentous fiber identical to that seen in our case.^1^ Notably, they identified it during the procedure on intracardiac imaging. This suggests that silicone–PTFE polymer liners, while providing low friction, may be susceptible to delamination under specific mechanical stresses during catheter exchanges through the sheath.

Before the introduction of Octaray™ (8 Fr outer diameter, 8 splines, and 48 electrodes) in 2019, PentaRay™ (7 Fr, 5 splines, and 20 electrodes) was universally used for arrhythmia mapping in conjunction with the Vizigo™ sheath, which has an inner diameter of 8.5 Fr. Delamination of the CARTO Vizigo™ sheath has not been reported with the use of PentaRay™. Octaray™ has essentially replaced PentaRay™ for obtaining quick high-resolution maps during catheter ablation. It is possible that a larger French size of Octaray™ with an additional two splines may cause more friction and shear stress on the inner lining of the CARTO Vizigo™ sheath, making it susceptible to delamination. We experienced a small but appreciable resistance with Octaray™ compared to the PentaRay™ catheter during catheter exchange through the CARTO Vizigo™ sheath. Damage to the valve of the Vizigo™ sheath during introduction of mapping or ablation catheter may also be a stress point for delamination during catheter exchange. Folding of the splines upon the catheter shaft within the sheath during introduction and/or advancement of the catheter can cause significant shear stress on the inner lumen of the sheath and predispose to delamination. Although imaging with ICE can identify the delamination, use of intermittent fluoroscopy may allow early identification of delamination given the fabric of the sheath is radiopaque.

To summarize, delamination of the CARTO Vizigo™ sheath is a real-world potential complication that can arise during ablation procedures. This complication may predispose to thromboembolic consequences. The resolution of symptoms in our case was more consistent with a small thromboembolism rather than a fabric embolism, which would have resulted in a larger stroke and likely a permanent deficit. Our report emphasizes the importance for proceduralists to be vigilant regarding device integrity during catheter ablation. It is imperative to examine the sheath carefully for manufacturing defects before, during, and after use. Damage to the inner lining of the sheath with subsequent delamination can be prevented or minimized by careful introduction (especially through the valve) and advancement of the mapping and ablation catheters through the sheath and minimizing catheter exchanges. Difficulty or resistance at the time of catheter introduction and/or advancement through the sheath should alert operators about this potential complication. A foreign material on sheath or catheters on ICE imaging may suggest a delamination process. If detected, efforts should be made to aspirate and extract the material and to carefully exchange and examine the sheath. Such events warrant immediate reporting to the manufacturer for analysis.

## Data Availability

The data involved in this case are available from the authors upon request.

## References

[1] Gatto MC, Verticelli L, Lumia G, Bellini C, Gaspardone A, Lamberti F (2025). First reported case of multielectrode mapping catheter delamination detected by intracardiac echocardiography during atrial fibrillation ablation. HeartRhythm Case Rep.

[2] Skala T, Moravec O, Hutyra M, Tudos Z, Kocher M, Taborsky M (2019). Apparent foreign-body-like structure detected in the left atrium after catheter ablation for persistent atrial fibrillation. Biomed Pap Med Fac Univ Palacky Olomouc Czech Repub.

[3] Kasai Y, Haraguchi T, Kitai T (2024). Successful transvenous retrieval of an electrode ring dislodged from a novel visualized steerable sheath by inflation of a balloon inside the ring. J Cardiol Cases.

